# Post-COVID-19 Vaccine Limbic Encephalitis: A Case Report

**DOI:** 10.7759/cureus.29003

**Published:** 2022-09-10

**Authors:** Khalid Albsheer, Abdalla Fadul, ELMustafa Abdalla, Gihan Mohamed, Mohamed Elawad, Abdulaziz Zafar

**Affiliations:** 1 Internal Medicine, Hamad General Hospital, Doha, QAT; 2 Internal Medicine, Hamad Medical Corporation, Doha, QAT; 3 Radiology, Hamad General Hospital, Doha, QAT

**Keywords:** limbic, encephalitis syndrome, sars-cov-2-associated encephalitis, autoimmune limbic encephalitis, covid-19 encephalitis

## Abstract

Limbic encephalitis (LE) diagnosis can be challenging due to its broad spectrum of clinical presentation and variety of causes. The most commonly known causes include paraneoplastic and autoimmune, but they can also occur post-vaccine. Since 2020, many people worldwide have received the coronavirus disease 2019 (COVID‐19) vaccine after FDA approval. Mild self-limited neurological adverse reactions, including headache and dizziness, were reported post-vaccine. However, emerging few neurological severe events, including encephalitis, have also been reported. Herein, we present a case of a middle-aged female who presented with seizures after two days of receiving the second dose of the Moderna COVID-19 vaccine. A diagnosis of limbic encephalitis was made based on head MRI findings. It was treated with immunosuppressive agents and responded well with no additional neurological sequelae. This case is unique as it highlights a possible association between limbic encephalitis and the COVID-19 vaccine.

## Introduction

Severe acute respiratory syndrome coronavirus 2 (SARS‐CoV‐2) started in December 2019 and caused the coronavirus disease 2019 (COVID‐19). COVID-19 is a systemic disease that can lead to numerous complications affecting almost every system in the body. Various types of COVID-19 vaccines have been distributed worldwide; moreover, despite the latest studies on these vaccines and their safety, numerous registered complications can be related to the vaccine [1].

Limbic encephalitis (LE) is an acute condition of noninfectious inflammation of the brain that affects the limbic system (hippocampus, medial temporal lobe, cingulate cortex, and frontonasal cortex). Clinical manifestations include amnesia, behavioral changes, psychiatric symptoms, seizures, and disturbed level of consciousness [2].

The pathophysiology of LE is known to be mediated by an antigen that stimulates an antibody-mediated host immune response that inadvertently targets self-antigens in the limbic area, which includes the cingulate cortex, frontonasal cortex, hippocampus, and medial temporal lobe [3].

This disease generally has two causes: paraneoplastic and autoimmune. The paraneoplastic form is associated with certain tumors, including small cell lung cancer (SCLC), germ cell testicular tumor, breast cancer, Hodgkin’s lymphoma, immature teratoma, and thymoma [4].

For patients with presumed autoimmune or paraneoplastic encephalitis, the diagnosis should be based on the history, clinical features, laboratory and radiologic evidence of central nervous system inflammation, exclusion of infection, and other alternative causes. The workup should include neuroimaging, EEG, lumbar puncture (LP), and antibody testing on serum and CSF. Although antibody testing can confirm the diagnosis, the initiation of therapy should not be postponed while waiting for antibody results [5].

Herein, we report a case of acute encephalopathy after receiving the COVID-19 Moderna vaccine. An extensive workup was done, including a complete blood workup, brain imaging, and autoimmune profile, but all turned out to be negative.

## Case presentation

A 35-year-old female, previously healthy, was brought to the hospital after one seizure. Her symptoms started two days post-Moderna COVID-19 vaccine second dose. Initially, she had a fever, subsequently followed by generalized tonic-clonic seizures that lasted for approximately five minutes and a post-ictal phase of confusion for half-hour. During her stay in the emergency department, she again developed two episodes of generalized seizures, controlled with medication, each one lasting less than two minutes after intervention. The patient did not have any other associated neurological symptoms. The patient was admitted to the ward and was vitally stable and afebrile. Apart from looking tired, she was oriented to time, place, and person. Her neck was supported, and she had full power and sensation with normal higher functions. Cranial nerves were intact. The patient had a full basic blood workup, which included a complete blood count and renal and liver function tests, which were all unremarkable, and her COVID-19 PCR was also negative. Surprisingly, her contrast MRI on admission was also unremarkable. LP was performed, and CSF had only significant lymphocytosis; other CSF workups, including viral panel and tuberculosis workup, were negative.

**Figure 1:**
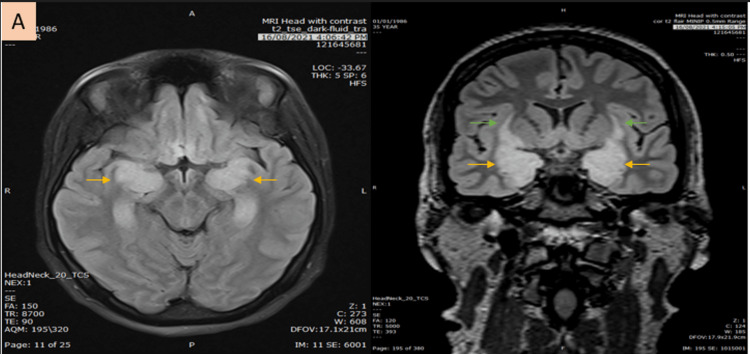
Axial and coronal head MRI T2/FLAIR show symmetrical swollen bilateral hippocampal (yellow arrow) and claustrum (green arrow) with T2/FLAIR bright signal. The same regions of both hippocampi and claustrum demonstrate T1 relatively low signal and no significant contrast enhancement (not shown). MRI: magnetic resonance imaging; FLAIR: fluid-attenuated inversion recovery

**Figure 2:**
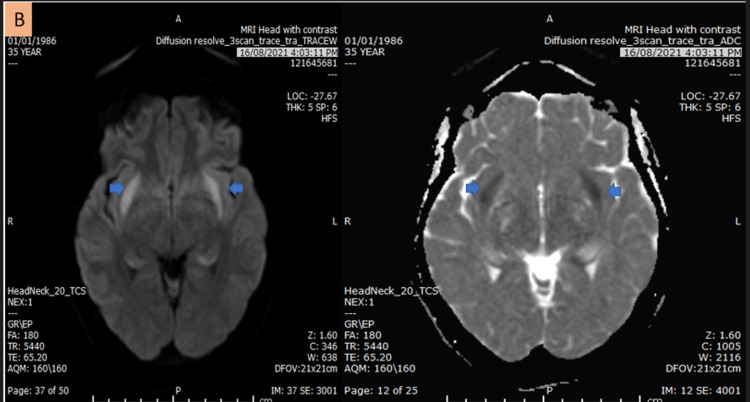
Head MRI axial diffusion and ADC map demonstrate symmetrical claustrum diffusion restriction (blue arrow). MRI: magnetic resonance imaging; ADC: apparent diffusion coefficient

During her hospital stay, she developed anisocoria. An urgent CT of the head showed possible temporal lobe hypodensities. After discussion with the radiologist, she agreed to repeat the head MRI (Figure 1 and Figure 2), which this time showed features suggestive of limbic encephalitis (paraneoplastic and autoimmune types). The differential diagnosis of the MRI findings included viral (herpes encephalitis), post-ictal, and Hashimoto’s encephalopathy. Subsequently, an EEG was done, which showed left frontotemporal epileptiform discharges with severe post-ictal encephalitic changes.

Meanwhile, the results of the immunology screen became available and showed positive antinuclear antibody (ANA), centromere pattern with 1:1280 and anti-centromere antibody, negative anti-dsDNA, and no hypocomplementemia. Immediately, the neurology team assessed the patient and started on steroids, IV immunoglobulin, and rituximab and planned for a repeat LP to check for autoimmune encephalitis, which unfortunately was not done due to financial issues. While the patient was still admitted, she developed abnormal behavior in the form of agitation, abnormal laughing, and crying, which is a well-known association with limbic encephalitis, which improved later with therapy. The patient was diagnosed with autoimmune limbic encephalitis that resulted in focal seizures with secondary generalization based on clinical presentation, imaging, LP finding, and negative workup for malignancy, including a CT scan of the neck, chest, abdomen, and pelvis, along with a PET scan. The patient started to improve day by day and was transferred to a rehabilitation facility to achieve full recovery. Neuroimaging follow-up showed improvement and resolution of the previous limbic encephalitis changes.

## Discussion

Autoimmune limbic encephalitis is a wide range of neurological diseases characterized by the formation of autoantibodies against neuronal cell surface antigens [6]. Apart from the typical encephalitis symptoms of altered cognition, fever, and focal neurological abnormalities, limbic encephalitis can also cause neuropsychiatric features.

Thus, a complex set of investigations is required before the official diagnosis [7]. Limbic encephalitis can be caused by various factors, including paraneoplastic, autoimmune, and post-viral diseases [8]. However, nontypical etiologies such as post-vaccination types, as described in very few documented post-vaccination cases, including the COVID-19 vaccine, are not rare [9]. In a survey conducted in the United States over 20 years, 141,396 incidences of encephalitis were reported, followed by vaccination of hepatitis B (354 cases), influenza (208 cases), measles, mumps, and rubella (MMR) (208 cases), and *Haemophilus influenzae* type B (208 cases). Vaccinations were all implicated (120 cases in 708 cases, and the development of encephalitis occurred within two weeks of vaccination (50.7%)).

As a result, the temporal relationship between vaccination and symptom start is consistent with our case [10]. Vaccine-related encephalitis pathophysiology is currently unknown and based mainly on speculation. On the other hand, vaccines are well known for triggering the generation of pro-inflammatory cytokines and T-cell response.

Antigens will be recognized as possible pathogens by the immune system after immunization. The immune system initiates a sophisticated series of natural immunological processes based on the immunogenetic background and innate immune memory, releasing mediators and products of inflammation into the circulation, which can cause systemic side effects and, in rare cases, neuroinflammation [11].

We believe that this episode of limbic encephalitis, which occurred quickly after COVID-19 vaccination, is an uncommon side effect of the vaccine. Our theory is based mainly on the timing of the symptoms, which began a few days after the immunization and in the absence of another identifiable cause. The patient had an immune system-activating immunization and experienced an unusual autoimmune reaction shortly afterward. There is, however, some skepticism, mainly because the patient’s symptoms and signs have yet to be appropriately linked to a cause.

## Conclusions

Autoimmune limbic encephalitis is challenging to diagnose for many reasons, one of which is that the clinical presentation can mimic numerous other diseases, and therefore, the differential diagnosis is broad. In addition to that, radiological manifestations are frequently absent or nonspecific in the more common subtypes. Being aware of the condition and how it manifests makes it one of the possible differential diagnoses in undifferentiated neuropsychiatric presentations, which will improve treatment outcomes in return.
